# The Effects of Milk and Dairy Products on Sleep: A Systematic Review

**DOI:** 10.3390/ijerph17249440

**Published:** 2020-12-16

**Authors:** Yoko Komada, Isa Okajima, Tamotsu Kuwata

**Affiliations:** 1 Faculty of Liberal Arts, Meiji Pharmaceutical University, 2-522-1 Noshio, Kiyose, Tokyo 204-8588, Japan; 2 Department of Psychological Counseling, Faculty of Humanities, Tokyo Kasei University, 1-18-1 Kaga, Itabashi-ku, Tokyo 173-8602, Japan; okajima-i@tokyo-kasei.ac.jp; 3 International Life Sciences Institute Japan, 3-5-19 Kojimachi, Chiyoda-ku, Tokyo 102-0083, Japan; kuwata@human.ac.jp; 4 Department of Health Food Sciences, University of Human Arts and Sciences, 963-9 Yamaguchi Tokorozawa, Saitama 359-1145, Japan

**Keywords:** sleep, milk, dairy product, human, systematic review, study quality

## Abstract

Several studies have assessed the effects of milk and dairy product intake on sleep quality and duration. Such investigations have varied in terms of their geographic locations, amounts of milk and dairy products, study participants (age, sex, race), and study designs. The present study aimed to summarize this literature and provide a unified view on whether the intake of milk and dairy products affects sleep quality. This systematic review was conducted according to the preferred reporting items for systematic reviews and meta-analyses (PRISMA) guidelines. The following keywords were chosen as electronic database search items from MeSH (medical subject headings) terms and descriptors in health sciences (DeHS) lists: milk, yogurt, dairy product, cheese, sleep, human, observational study, and interventional study. As a result, a total of 14 studies published between 1972 and 2019 were included in this review, including eight randomized controlled trials, two experimental studies with cross-over designs, one longitudinal study, and three cross-sectional studies. Four studies targeted older adults, three included toddlers, two targeted children, and six enrolled adults inclusive of university students. Overall, these studies indicated that a well-balanced diet that includes milk and dairy products is effective in improving sleep quality, despite mixed results across studies attributable to differences in study populations and methods.

## 1. Introduction

The intake of milk and dairy products is generally considered to promote good sleep quality and to have a positive effect on physical and mental health. As for the mechanism of action, it is believed that a high amount of tryptophan (Try), from which melatonin is synthesized, contained in milk and dairy products can suppress the action of the inhibitory neurotransmitter gamma-aminobutyric acid (GABA) [1]. The anti-inflammatory effects of milk and dairy products, due to their containing antioxidant and anti-inflammatory components, as well as activity changes within the brain-gut-microbiome axis, has recently garnered extensive attention in the literature [2].

Blood melatonin levels are synchronized by circadian rhythms, and this hormone induces sleep onset in humans. Melatonin is synthesized in vivo from Try via serotonin synthesis. The α-lactalbumin protein contained in milk has the highest Try content among the food proteins normally consumed by humans. Furthermore, the amino acid composition of α-lactalbumin allows Try to cross the blood-brain barrier (BBB) [3]. As the BBB transporter is shared by the large, neutral amino acid LNAA (tryptophan (Try), phenylalanine (Phe), valine (Val), leucine (Leu), isoleucine (Ileu), tyrosine (Tyr)), Try transfer into the brain is determined by the Try/LNNA ratio. An increased amount of Try passes through the BBB when blood glucose concentration is high. Therefore, beverages in which milk has been sweetened with sugar additives (e.g., malted milk products such as Horlicks) have been produced and are now being advertised and marketed as sleep aids [3].

Although some studies have focused on the antioxidant components and anti-inflammatory effects of milk products [4], it is not easy to directly explain the relationship between sleep quality and dairy intake because it may be masked by the much greater effect of diet as a whole. Recently, attention has been focused on the intestinal microbiota and its relation to sleep via the modulation of the brain-gut-microbiome axis [5]; however, since the whole diet modifies the intestinal microbiota, interventional studies have not yet been conducted to assess dairy products alone.

GABA contained in fermented dairy products is a non-protein amino acid produced from glutamic acid via decarboxylation catalyzed by the enzyme glutamine decarboxylase, produced by lactic acid bacteria [6]. GABA is an inhibitory neurotransmitter in the central nervous system of mammals synthesized in the body during sleep, but disruptions of the sleep cycle and stress-induced deterioration of sleep quality reduces the amount of GABA synthesized, further impeding sleep onset [7,8]. Foods rich in GABA include fermented foods such as kimuchi, natto, and yogurt, as well as germinated brown rice and potatoes. GABA is presumed to be involved in the improvement of sleep quality observed upon the ingestion of fermented dairy products.

As a result of the identification of these mechanisms of action, various studies have been conducted to better assess the effects of milk and dairy product intake on sleep quality and sleep duration. However, due to differences in research methodologies, such as the amount of milk and dairy products ingested, the participants’ race, sex, and age, and intervention techniques, whether milk and dairy consumption have a positive effect on sleep remains unclear. In addition, whether any improvement in overall sleep is the result of better sleep quality, longer sleep duration, or improvements in sleep-wake rhythms also remains vague. Thus, it is necessary to comprehensively consider the relationship between the intake of milk and dairy products and sleep. The purpose of this study was to clarify these points through a systematic review.

## 2. Materials and Methods

### 2.1. Protocol

This systematic review was conducted and reported according to the preferred reporting items for systematic reviews and meta-analyses (PRISMA) guidelines [9]. This study was funded by the Japan Dairy Association.

### 2.2. Literature Search

Information pertaining to the characteristics of population/patient, intervention, comparison/comparators and outcomes (PICOS) criteria adopted by the selected articles in this study are shown in Table 1. Three authors (TK, IO, and YK) independently searched for original articles that investigated the effects of milk products on sleep, using electronic databases, including MEDLINE (PubMed, www.pubmed.com). Keywords were chosen from MeSH (medical subject headings) terms and descriptors in health sciences (DeHS); the following search terms were used (Table 1): milk, yogurt, dairy product, cheese, sleep, human, observational study, and interventional study. The final search was conducted on 20 August 2019.

### 2.3. Article Selection Process

The study selection was performed by three authors (TK, IO, YK) in the following three analyses phases: titles, abstracts, and full-text analyses. The present systematic review excluded comment articles, review articles, letters, case reports, abstracts, and unpublished articles. We also excluded studies on breast-fed babies.

### 2.4. Data Extraction

After reading the selected studies, a comparison of all compiled data was conducted by the authors (TK, IO, and YK) to verify their integrity and reliability. Differences in decisions regarding selected studies were settled by consensus. For each included study, the following information was extracted: title, author name(s), year of publication, study purpose, subjects’ characteristics, sample size, study design, intervention, and main results.

### 2.5. Risk of Bias: Assessment of Study Quality

A modified Jadad scale [10] was used to evaluate the quality of the retrieved randomized controlled trials (RCTs). Each study was assigned a quality score between 0 and 5 based on the quality of the randomization, the method of generation of random numbers, details describing double-blinding procedures, information about withdrawals, and allocation concealment. High-quality RCTs scored ≥3 points, whereas low-quality RCTs scored <2 points, according to the modified Jadad score.

The Newcastle-Ottawa scale (NOS) was used to evaluate the overall quality of observational studies [11] by totaling scores based on the following nine criteria (0 to 9 stars): representativeness of the exposed cohort, selection of the non-exposed cohort, ascertainment of exposure, and whether the outcome of interest was present at the beginning of the study (maximum of 4 stars), comparability of the cohorts on the basis of study design and analysis methodology (maximum of 2 stars), and assessment of outcome measures (maximum of 3 stars). Studies with scores of ≥6, 4–5, and 0–3 were defined as being of high-, moderate-, and low-quality, respectively, as shown in Table 2.

### 2.6. Data Analyses

All 14 studies reviewed in this article are summarized in Table 2 according to their main characteristics and findings concerning sleep-associated variables. The studies are organized chronologically by year of publication, starting with the earliest published study.

## 3. Results

### 3.1. Study Selection

**Figure 1:**
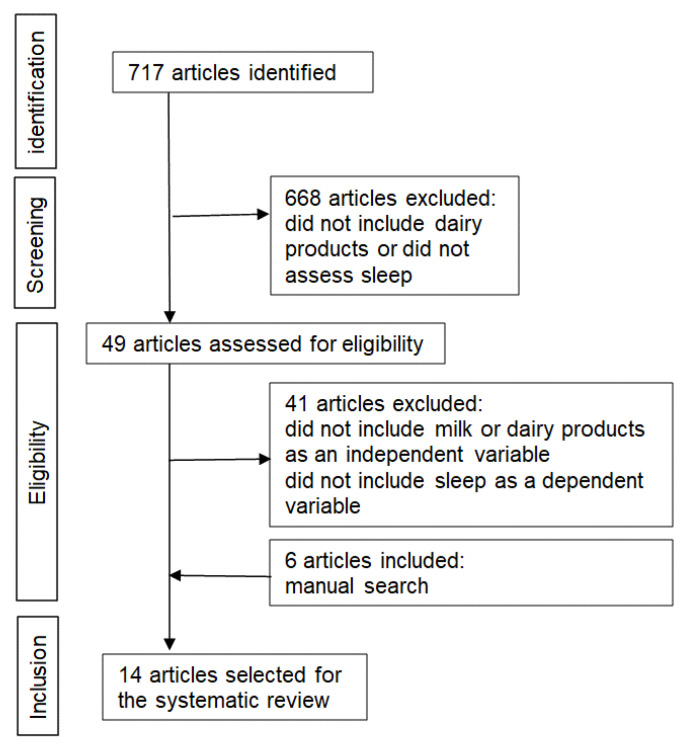
Flowchart of the study selection process.

PubMed database searches identified 717 studies. Of these, 668 were excluded based on their titles since they were considered irrelevant to the topic of interest (first screening). After reading the abstracts of the identified reports, the following studies were selected: (1) those with milk product consumption as independent variable and (2) those with sleep variables as dependent variables (second screening). Three authors (TK, IO, and YK) confirmed and discussed the articles’ content (third screening). Eight of these met all the criteria for the systematic review [12,13,14,15,16,17,18,19]. Additional studies were manually identified through other sources (*n* = 6) [20,21,22,23,24,25]. The reasons for the inclusion/exclusion of studies are shown in Figure 1.

### 3.2. Description of Included Studies

A total of 14 studies published between 1972 and 2019 were included in this review [12,13,14,15,16,17,18,19,20,21,22,23,24,25]. In these studies, sample sizes ranged from 4 [15] to 4552 [23], with a total of 10,833 participants (Table A1).

Four studies targeted older participants [12,14,19,25], three included toddlers [13,18,20], two targeted children [16,17], and six targeted adults, including university students [14,15,21,22,23,24].

The geographical areas covered in the studies were North America (three studies) [13,17,23], Oceania (one study) [16], Europe (five studies) [12,14,15,18], Japan (four studies) [19,22,24,25], and Asia other than Japan (one study) [20]. The research design of these studies included RCTs (*n* = 8), experimental studies such as cross-over trials (*n* = 2), a longitudinal study (*n* = 1), and cross-sectional studies (*n* = 3).

The independent variables were Try concentration (*n* = 3), milk consumption at night (*n* = 1), Horlicks consumption (*n* = 2), fermented milk consumption (*n* = 2), high or low glycemic index (GI) (*n* = 2), and the proportion of consumed milk product (*n* = 2). One study that assessed Try showed a comparison of the effect of different quantities of Tyr on sleep: 892 μmol/L, 1192 μmol/L, 1513 μmol/L, 1808 μmol/L [13]. The second Try study assessed the effect of consuming Try-enriched milk (3.4 g Try/100 g protein) during nighttime compared to controls who consumed regular milk (1.5 g Try/100 g protein) [18]. The third study compared Try-rich alpha-lactalbumin or placebo protein with low Try content [21]. One of the studies assessing Horlicks consumption compared 32 g of Horlicks combined with 250 mL of hot milk or control milk [14]; the other compared 350 mL of warm water and 350 mL of warm milk combined with Horlicks [15]. One of the studies that assessed fermented milk consumption compared fermented and artificial milk [19]; the other compared fermented milk containing the *Lactobacillus casei* strain Shirota (LcS) and non-fermented, placebo milk (a daily dose of 100 mL) [24]. One of the studies assessing GIs compared a high GI (200 mg low fat cow’s milk with 50 g Glucodin powder) and a low GI drink (200 mg full cream cow’s milk with 50 g honey) [16]; the other compared low GI (23) milk and high GI (65) milk [20].

### 3.3. Main Results of the Studies

Although the extents of the effects of dairy on characteristics of sleep differed somewhat between studies, several studies observed shorter sleep latency, less waking after sleep onset (WASO), greater sleep efficiency, longer sleep duration, and a lower awakening index in those consuming high Try-enriched milk relative to low-Try milk [13,18,21]. Milk consumption at night did not improve sleep quality [12]; this may be attributable to the differences in the amounts of tryptophan and duration of intake compared to the control condition. In a study of healthy college students, only one trial for two days was conducted, and whether the study controlled for dietary intake other than milk consumption was unclear [21].

Based on the results of the cross-sectional studies, the combination of engaging in physical activity and the consumption of milk or cheese is necessary to improve the ability to fall asleep in older adults [25]. Among younger adults, participants with a late chronotype consumed fewer milk products compared to those with an early chronotype [22]. The 2007–2008 National Health and Nutrition Examination Survey revealed that low calcium intake was associated with difficulty falling asleep and an increase in non-restorative sleep [23]. In a longitudinal study, girls with a short persistent/increasing sleep pattern consumed milk products significantly less often (≤once per day) and drank soft drinks significantly more often (≥once per day) than girls with 10-h persistent or 11-h persistent sleep patterns [17].

Subsequently, we reviewed the effects of milk product consumption on sleep with the participants stratified by generation. Among toddlers, sleep latency was shortened in those consuming milk products with added Try [13]. There were no significant differences in the latency of sleep onset, total sleep time, WASO, or sleep efficiency between 14–24-month-old toddlers consuming high or low GI formulas across a period of 3.5 days. Low GI formulations are preferable because they tend to be high in dietary fiber and low in refined sugars and starches [20]. Among school-aged children, the mean total arousal index in the first half of the night was greater after consuming beverages with a high GI for one day than a low-GI drink, and the arousal index during non-rapid eye movement (NREM) sleep in the first half of the night was higher after consuming a high-GI drink than a low-GI drink [16]. It has been suggested that Horlicks may improve the sleep of younger individuals [14,15]. In addition, Horlicks combined with hot milk improved sleep among older participants [14]; however, the effectiveness of night milk on sleep is inconsistent among older participants [12]. Participant age group, intervention, main results and implications of each study are summarized in Table 3. Although the age range of the participants (children, adults, the older adults), interventions (dairy products intake, nutritive component of dairy products, dietary habits) varied across studies, several positive effects on different sleep outcomes (electroencephalography, actigraphy, and subjective assessment) were reported.

### 3.4. Study Quality

We assessed 10 clinical studies and four observational studies using the modified Jadad scale and the NOS. Among the 10 clinical studies, three were determined to be of high quality; the other seven were determined to be of low quality. Of the four observational studies, the NOS classified one as being high quality, one as moderate quality, and two as low quality.

## 4. Discussion

The present systematic review was performed to determine the relationship between milk and dairy product consumption and sleep by summarizing the methodologies and results of studies that met our selection criteria. Eight of the selected studies were chosen based on automated PubMed searches [12,13,14,15,16,17,18,19], and six studies were manually selected [20,21,22,23,24,25]. The studies varied in terms of their geographic locations, amounts of milk and dairy products considered, study participants, and study designs. Several positive effects of milk and dairy products on different sleep outcomes were revealed (electroencephalography, actigraphy, and subjective assessment); however, the interpretation of such findings requires caution because the age range of the participants (children, adults, the elderly) and interventions (dairy products intake, nutritive component of dairy products, dietary habits) varied across the studies (Table 3). While RCTs investigating milk intake at night, consumption of Horlicks milk containing added Try, and fermented milk all showed partial, positive effects on sleep quality (sleep onset latency, WASO, sleep duration) [12,13,14,15,18], the effects were limited, and any positive effects were considered to be small. Such limited results may also be due to the difficulty of controlling for long-term conditions in human intervention trials.

Cross-sectional study has suggested relationships between high milk intake, ease of falling asleep, and an early chronotype [22]. The circadian rhythm is regulated by the body clock, which is largely influenced by light exposure and diet. By eating food rich in Try at breakfast and exposing oneself to light during the daytime, the onset of nighttime melatonin secretion could be accelerated [26]. Melatonin synthesized from tryptophan via serotonin is known to induce sleep onset in humans [27]. The relationship between the amount of tryptophan intake and sleep improvement requires elucidation. In a survey of elderly Japanese participants, a combination of high physical activity and intake of milk and dairy products was associated with good sleep quality [25]. Thus, it may be expected that a person with healthy eating habits and overall lifestyle that includes physical activity will experience good sleep quality. To the best of our knowledge, the literature features no reports concerning the effect of milk and dairy product on sleep in lactose-intolerant individuals. Lactose intolerance should be diagnosed by a lactose intolerance test, but at an early age, after drinking milk, people experience symptoms such as abdominal distention, flatulence, and diarrhea, which causes dislike of milk. It has been reported that even those who avoid milk intake can reduce their digestive symptoms by continuing to consume fermented milk and cheese with low lactose content, considering the nutritional benefits of milk and dairy products and the variety of dietary contents [28]. Such individuals should be considered in future studies.

Antioxidant and anti-inflammatory components in milk and dairy products may also contribute to improvements in sleep quality. A cross-sectional study of older participants [29] and middle-aged adults [30] reported that a Mediterranean diet had a positive effect on sleep quality. Older adults who frequently consumed many vegetables, whole grains, legumes, fruits, olive oil, and seafood slept better than their counterparts who did not, suggesting the importance of the food intake in sleep quality. The authors noted that the major ingredients of the Mediterranean diet are rich in antioxidants and anti-inflammatory substances that help to suppress inflammation in the body [29]. Furthermore, milk and dairy products contain more antioxidants of low molecular weight (various vitamins, nitrogen-containing components, and peptides such as glutathione) and anti-inflammatory proteins (lactoferrin, lactoperoxidase, lactalbumin, etc.) compared to other animal-derived foods. Finally, a Mediterranean diet with frequent milk and dairy product consumption was suggested to be effective in reducing the risk of developing dementia in older Japanese adults when combined with exercise and good dietary habits [31].

As the present systematic review included a variety of study participants, including infants, children, young adults, and older adults, we further explored whether the effects of dairy on sleep varied with age. For example, infants often experience difficulty in falling asleep and frequent awakenings during sleep. Two studies have suggested that fortifying milk with Try improves sleep quality and increases sleep duration [13,18]. When comparing nighttime milk consumption with commercial milk, the effect was inconsistent, rendering it difficult to base any conclusions on the findings. Two studies, however, have shown that malted drinks (such as Horlicks) and fermented milk can improve sleep [14,15]. Study of older adults have assessed the impact of dairy intake on problems such as poor sleep quality, awakening during sleep, and decreased physical activity during the day [12]. It seemed hard to conclude the effect of night milk on sleep quality among older adults, because both night milk and normal milk improved the quality of sleep in some subjects, while in others it did not change. Since older people are more likely to have underlying medical conditions, it is presumed that the difference of medical condition changes the effect of milk.

Daytime physical activity is also important for improving nighttime sleep, but only one study has examined this association [12]. The ingestion of milk and dairy products reportedly restores physical and mental functioning and increases daytime activity [32], which may contribute to better sleep at night. Therefore, it is necessary to consider not only the direct effects of dairy product intake on sleep, but also the indirect effects resulting from increased physical functionality during the day.

This study is subject to several limitations. First, stress-related mental health and depressive symptoms were not included in the search terms. Stress is closely related to sleep quality [33,34], and both variables should be considered. Second, the present study did not use the specific ingredients in milk such as Try, GABA, and melatonin as the search terms. While this omission reflected our aim of revealing the effects of consuming milk and dairy products on sleep rather than the effects of specific ingredients found in dairy on sleep, future studies should elucidate the relationships between the ingredients in dairy products and sleep and thereby improve the current understanding of their mechanism of action. Third, the sample sizes of the included studies were relatively small, particularly those of the longitudinal studies. There were also variations in the outcomes across studies. Hence, the generalization of these findings requires caution. We refrained from conducting a meta-analysis to avoid the so-called “garbage in, garbage out” problem. Both meta-analyses and further experimental studies will be needed to clarify the long-term efficacy of milk and dairy products in improving sleep. In addition, only a few studies included in this review were determined to be of a high quality. More studies of great rigor and quality may be warranted to determine the relationship between dairy intake and sleep quality.

## 5. Conclusions

The present systematic review of past studies published between 1972 and 2019 aimed to assess the relationship between milk and dairy product intake and sleep. We observed that a well-balanced diet that includes milk and dairy products is considered to be effective for improving sleep quality, although the results of some of the studies were mixed and their conclusions limited as a result of small sample sizes and poor study quality. According to dietary intake standards in the United States, milk and dairy products are classified as foods with high nutritional density. People who consume adequate amounts of milk and dairy products meet the recommended requirements for daily calcium intake, and such people tend to have high health literacy and better sleep habits. In order to maintain good quality sleep, it is important to consider all lifestyle habits, including the consumption of healthy meals. Milk and dairy product consumption in the diet could be an index of sleep quality.

## Figures and Tables

**Table 1:** PICOS1 criteria for inclusion in the review and search terms.

Parameter	Inclusion Criteria
Participants	Human
Intervention or exposure	Consumption of milk, yogurt, cheese, or other dairy products
Comparator(s)	–
Outcome	Sleep
Study type	Observational study, intervention study
#1. milk [title/abstract]	#6. sleep [title/abstract]
#2. yogurt [title/abstract]	#7. human [text word]
#3. “dairy product” [title/abstract]	#8. “observational study” [text word]
#4. cheese [title/abstract]	#9. #5 and #6 and #7 and #8
#5. #1 or #2 or #3 or #4	

1 PICOS = participant, intervention, comparator, outcome, study type.

**Table 2:** Risk of bias: assessment of study quality of articles included in the review.

Study (Year)	Newcastle-Ottawa Scale (NOS) Scoring				Jadad Scale Scoring			
	Selection	Comparability	Outcome	Total Score	Randomization	Double-Blinding	Withdrawals and Dropouts	Total Score
Valtonen et al. (2005)					no	yes	yes	2
Steinberg et al. (1992)					yes	yes	no	2
Brezinova et al. (1972)					no	no	no	0
Southwell et al. (1972)					no	no	no	0
Jalilolghadr et al. (2011)					yes	no	no	1
Aparicio S et al. (2007)					no	yes	no	1
Yamamura et al. (2009)					yes	yes	yes	3
Misra et al. (2015)					yes (extra point added)	yes (extra point added)	no	4
Markus et al. (2005)					no	yes (extra point added)	no	2
Takada et al. (2017)					yes (extra point added)	yes (extra point added)	yes	5
Tatone-Tokuda F et al. (2012)	★★★	★	★★★	7				
Sato-Mito et al. (2011)	★★	★		3				
Grandner et al. (2014)	★★★	★		4				
Kitano et al. (2014)	★★	★		3				

★★★ high-quality, ★★ moderate-quality, ★ low-quality.

**Table 3:** Systematic review summary.

Intervention	Category	Participant Age Group	Main Results	Implications
Night milk	Dairy product intake, RCT [12]	Older people	Positive and negative effects on sleep from subjective measurement	Effectiveness is inconsistent for older people with and without underlying diseases
Fermented milk	Dairy product intake, RCT and non-RCT [19,24]	Older people	Positive effects on sleep (sleep efficacy, wake episodes on actigraphy) from objective measurement	Certain effects on sleep have been reported
		University students	Positive effects on sleep (sleep latency and stage 3 non-REM sleep measured by EEG, subjective sleep length) from objective measurement	
Milk plus Horlicks	Nutrients, RCT [14,15]	Adults	Positive effects on sleep (restlessness during sleep, total sleep duration and wake episode measured by EEG) from objective measurement	Certain effects on sleep have been reported
		Older people	Positive effects on sleep (small movements during sleep) from objective measurement	
Tryptophan	Nutritive component of dairy products, RCT and non-RCT [13,18,21]	Infants	Positive effects on sleep (observational sleep latency, quiet sleep, active REM sleep) from subjective measurement	Certain effects on sleep among infants, but no positive effects on sleep among university students have been reported
		Infants	Positive effects on sleep (sleep length, sleep efficacy on actigraphy) from objective measurement	
		University students	No positive effects on sleep. Evening consumption improved early morning performance from subjective and objective measurement	
High glycemic index milk	Nutrients, RCT and non-RCT [16,20]	Toddlers	No positive effects on sleep (sleep-onset latency, total sleep time, wake after sleep onset, sleep efficiency on actigraphy) from objective measurement	High GI milk has negative effects or no positive effects on sleep compared to low GI milk
		Children	Negative effects on sleep (arousal during sleep measured by polysomnography) from objective measurement	
Proportion of milk consumption	Dietary habit, observational study [17,22,23,25]	Children	Significant association between milk consumption and sleep (subjective sleep length) among girls	Cross-sectional and longitudinal studies suggested the relationship between proportion of milk and dairy products consumption and sleep
		University students	Significant association between less intake of milk and milk products and late midpoint of sleep	
		Adults	Significant association between low calcium intake and sleep (difficulty falling asleep and non-restorative sleep)	
		Older people	Significant association between dairy products consumption plus physical activity and sleep (subjective sleep latency)	

RCT: randomized controlled trial, EEG: electroencephalography, GI: glycemic index, REM: rapid eye movement.

## Appendix A

**Table A1:** Studies conducted to assess the effect of dairy products on sleep.

Study (Year)	Enrolled Subjects	Study Design	Country	Independent Variable	Dependent Variable	Results
Valtonen et al. (2005) [12]	Study 1: 59 females and 11 males (mean age = 81 ± 9 years, range = unclear); Study 2: 64 females and 17 males (mean age = 82.8 ± 8.1 years, range = unclear)	Study 1: Randomized controlled trial (RCT) (2-arms); Study 2: RCT (3-arms)	Finland	“Study 1: Group I: night milk (experimental) for 8 weeks and normal commercial milk (placebo) for 8 weeks, with a washout period of 1 week; Group II: normal daytime milk for 8 weeks, then switched to night milk after the washout period; Study 2: Group III: night milk for 8 weeks and normal daytime milk for another 8 weeks, with a washout period of 1 week; Group IV: normal milk for 8 weeks and night milk for another 8 weeks, with a washout period of 1 week. Group V: normal daytime milk throughout the experiment (control)”	Study 1: sleep quality, number of restless nights reported by nurses, Mini-Mental State Exam (MMSE) score; Study 2: sleep quality, daytime activity reported by caregivers	Study 1: decreased number of restless nights in Group I in Period 2 (normal milk), decreased sleep quality and number of restless nights in Group II in Period 2 (night milk); Study 2: increased sleep quality in Group III in Period 2 (normal milk). No effect of night milk. Increased sleep quality, morning activity and evening activity in Group IV in Period 2 (night milk). Slight increase in sleep quality In Group V in Period 2 (control).
Steinberg et al. (1992) [13]	57 infants	RCT (4-arms)	USA	Experimental formulae (EF): +0 (892 μmol/L tryptophan), +1 (1192 μmol/L tryptophan), +2 (1513 μmol/L tryptophan), +3 (1808 μmol/L tryptophan)	Sleep latency, quiet sleep, active rapid eye movement (REM) sleep (observation)	Shorter sleep latency in those fed with the higher tryptophan-containing formula.
Brezinova et al. (1972) [14]	Younger group: six males and four females (mean age = 22 years, range = 20–30); older group: three males and five females (mean age = 55 years, range = 42–66)	RCT (2-arms)	Edinburgh	32 g Horlicks solution with 250 mL of hot milk or control (inert yellow capsule)	Electroencephalography (EEG)	Younger group: diminished restlessness during sleep in Horlicks-administered group; older group: longer total sleep duration and fewer instances of awakening in the Horlicks group.
Southwell et al. (1972) [15]	Four male students (age: unclear)	RCT (2-arms)	London	No drink (control), 350 mL of warm water, or 350 mL of warm milk plus Horlicks	Movements during sleep	Horlicks consumption reduced the number of small movements during sleep
Jalilolghadr et al. (2011) [16]	Children (8–12 yrs; *n* = 8)	Experimental study	Sydney	High glycemic index (GI) drink (200 mg low fat cow’s milk with 50 g Glucodin powder) vs. low GI drink (200 mg full cream cow’s milk with 50 g honey) each for one day	PSG	The mean total arousal index in the first half of the night after the consumption of the high GI drink was greater than that of the low GI drink. (12.9 ± 4.6 vs. 9.9 ± 2.2, *p* = 0.03). Non-rapid eye movement (NREM) arousal index in the first half of night after the consuming the high GI drink was also higher than that of the low GI drink (12.7 ± 4.8 vs. 9.6 ± 2.3, *p* = 0.04).
Tatone-Tokuda F et al. (2012) [17]	Children (6 years old: *n* = 1106; 7 years old: *n* = 1015)	Quebec Longitudinal Study of Child Development (QLSCD) (longitudinal study: 1998–2010)	Canada	Proportion who had consumed a dairy product	Categorical sleep pattern characteristics (“short-persistent⁄increasing” sleep pattern, “10-h persistent,” “11-h persistent” patterns.	Girls who had a “short-persistent⁄increasing” sleep pattern consumed milk products significantly less often (≤once per day) and soft drinks significantly more often (≥once per day) than girls with “10-h persistent” or “11-h persistent” patterns. In boys or girls; milk products or soft drinks (in boys) at the 0.05 level (data not shown).
Aparicio S et al. (2007) [18]	Healthy infants (12–20 weeks of age; *n*-18) consuming artificial milk before the study	Experimental study (double-blind)	Spain (Balearic Island)	1. Control week: each infant received commercial infant milk throughout the 24 h of the day (1.5 g tryptophan/100 g protein). 2. Other control week (inverse): control milk from 18.00 to 06.00 h, and tryptophan-enriched milk (3.4 g tryptophan/100 g protein) from 06.00 to 18.00 h. 3. The experimental week the infants received control milk during light time (from 06.00 to 18.00 h) and tryptophan enriched milk during dark time (from 18.00 to 06.00 h)”	Sleep time; sleep efficacy; sleep bouts; immobility on activity recording	Slept longer, better sleep efficiency, more immobility during the experimental week.
Yamamura et al. (2009) [19]	29 healthy elderly subjects (aged 60–81 years)	RCT with double-blind, cross-over design	Japan	Fermented milk vs. artificial milk (placebo)	Sleep efficacy; sleep latency; waking episodes; waking after sleep onset (WASO) based on actigraphy	There was no significant difference between the groups. Sleep efficiency had improved significantly from the baseline period in those receiving the fermented milk (*p* = 0.03). The number of awakening episodes decreased significantly from the baseline period in the fermented milk group (*p* = 0.007).
Misra et al. (2015) [20]	56 toddlers (Age: 14–24 months)	A double-blind RCT (between subjects) design	Malaysia	Low GI (23) milk or high GI (65) milk for a period of 3.5 days	Sleep-onset latency (SOL), total sleep time (TST), WASO, sleep efficiency (SE).	“There were no significant differences between the two GI groups for SOL, TST, WASO, and SE. There was no need for any added sugars to improve the sleep patterns of this age group. The LGI formulations are preferable, as these tend to be higher in dietary fiber and lower in refined sugars and starches.”
Markus et al. (2005) [21]	Healthy college students with (*n* = 14) or without (*n* = 14) mild sleep complaints	A double-blind, placebo-controlled study	Netherlands	Tryptophan-rich alpha-lactalbumin (A-LAC) tryptophan-low placebo protein	The Stanford Sleepiness Scale, The Psychmotor Vigilance task, electroencephalographic recordings, concentrations of plasma tryptophan to other large neutral amino acids (Trp:LNAA)	The mean plasma Trp:LNAA was higher after the A-LAC diet condition than after the placebo diet condition (but not significantly). All subjects were less sleepy in the morning after evening intake of A-LAC than after placebo intake (but not significantly). Evening consumption of tryptophan-rich A-LAC may improve early morning performance indirectly through enhanced available brain tryptophan levels and subsequent sleep improvement.
Sato-Mito et al. (2011) [22]	3304 female dietetics students (Age: 18–20 years)	Cross-sectional survey	Japan	Midpoint of sleep	A validated 16-page self-administered diet history questionnaire (DHQ).	The late midpoint of sleep was significantly negatively associated with the percentage of energy derived from proteins and carbohydrates, and the energy-adjusted intake of cholesterol, potassium, calcium, magnesium, iron, zinc, vitamin A, vitamin D, thiamin, riboflavin, vitamin B6, folate, rice, vegetables, pulses, eggs, and milk and milk products.
Grandner et al. (2014) [23]	The 2007–2008 National Health and Nutrition Examination Survey (NHANES) (*n* = 4552)	Cross-sectional survey	USA	The 2007–2008 National Health and Nutrition Examination Survey (NHANES)”	“Sleep symptoms (difficulty falling asleep, difficulty maintaining sleep, non-restorative sleep, and daytime sleepiness)”	Low calcium intake was associated with difficulty falling asleep and a greater duration of non-restorative sleep.
Takada et al. (2017) [24]	94 medical students	Double-blind, placebo-controlled trial	Japan	*Lactobacillus casei* strain Shirota (LcS) or non-fermented placebo milk (a daily dose of 100 mL) for 8 weeks prior to and 3 weeks after the examination (under psychological stress)	Overnight single-channel electroencephalography (EEG) recordings, subjective sleep and anxiety reporting.	There was a significant positive effect of LcS treatment on subjective assessment of sleepiness on waking and sleep duration. Sleep latency measured by EEG increased as the exam approached in the placebo group but was significantly suppressed in the LcS group. The percentage of stage 3 non-REM (N3) sleep decreased in the placebo group as the exam approached, whereas it was maintained in the LcS group throughout the trial.
Kitano et al. (2014) [25]	421 community-dwelling older people aged ≥65 years (mean age: 74.9 ± 5.5 years; male: 43.7%)	Cross-sectional survey	Japan	The Pittsburgh Sleep Quality Index	Dairy consumption of habitual intake of milk, yogurt, and cheese; the Physical Activity Scale for the Elderly	The combination of engaging in leisure-time physical activity (LTPA) and consuming milk or cheese is necessary as a prescription to improve latency to sleep onset for older adults suffering from DIS.

RCT: randomized controlled trial, MMSE: Mini-Mental State Examination, EF: experimental formulae, REM: rapid eye movement, EEG: electroencephalography, GI: glycemic index, NREM: non-rapid eye movement, WASO: waking after sleep onset, SOL: sleep onset latency, TST: total sleep time, SE: sleep efficiency, Trp: tryptophan, A-LAC: alpha-lactalbumin, DHQ: Diet History Questionnaire, NHANES: National Health and Nutrition Examination Survey, LcS: *Lactobacillus casei* strain Shirota, LTPA: leisure-time physical activity.
